# Adjunctive therapeutic strategies in Obsessive-Compulsive Disorder resistant to serotonin reuptake inhibitors: a literature review

**DOI:** 10.1192/j.eurpsy.2022.1654

**Published:** 2022-09-01

**Authors:** P. Regueira, S. Hall, J. Cerejeira

**Affiliations:** 1 Centro Hospitalar e Universitário de Coimbra, Psychiatry, Coimbra, Portugal; 2 University of Coimbra, Faculty Of Medicine, Coimbra, Portugal

**Keywords:** obsessive-compulsive disorder, Treatment-resistant, Serotonin Reuptake Inhibitors, Adjunctive therapy

## Abstract

**Introduction:**

Obsessive-Compulsive Disorder (OCD) is a common mental disorder and a major cause of disability worldwide. Typically, it has a chronic course, marked by recurrent intrusive thoughts (obsessions) and repetitive behaviors (compulsions). Its pharmacological first line of treatment has been well established for several years now, with the Serotonin Reuptake Inhibitors (SRIs). However, about half of the patients are resistant to this approach, representing a therapeutic challenge for clinicians. Evidence suggests that other medications can augment SRIs, enhancing its effects and achieving a bigger efficacy in these patients’ treatment. Also, there is an increasing interest in neurosurgical interventions in these patients.

**Objectives:**

The main goal of this work was to assess the clinical efficacy of adjunctive therapeutic strategies in patients with OCD resistant to SRIs.

**Methods:**

A literature review was conducted searching PubMed and ScienceDirect databases from the 1st of January 2000 to the 1st of September 2021 to identify clinical trials comparing an active drug/neurosurgical intervention with placebo as an adjunctive therapeutic strategy in SRI-resistant OCD.

**Results:**

Sixteen studies were selected for data extraction, including a total of 585 patients. Risperidone, aripiprazole, N-acetylcysteine, lamotrigine, pindolol, riluzole, memantine and methylphenidate were efficacious for augmenting SRIs in OCD. Ablative surgery (ABL) and deep brain stimulation (DBS) were equal effective in the treatment of refractory OCD.

**Conclusions:**

Several therapeutic options presented as potentially effective in OCD when it is resistant to SRIs, although this is still an area for further research.

**Disclosure:**

No significant relationships.

